# Colectomy Rates for Ulcerative Colitis Differ between Ethnic Groups: Results from a 15-Year Nationwide Cohort Study

**DOI:** 10.1155/2016/8723949

**Published:** 2016-12-15

**Authors:** Ravi Misra, Alan Askari, Omar Faiz, Naila Arebi

**Affiliations:** ^1^St. Mark's Hospital & Academic Institute, Harrow, London HA1 3UJ, UK; ^2^Surgical Epidemiology, Trials and Outcome Centre (SETOC), St. Mark's Hospital & Academic Institute, Harrow, London HA1 3UJ, UK

## Abstract

*Introduction*. Previous epidemiological studies suggest a higher rate of pancolonic disease in South Asians (SA) compared with White Europeans (WE). The aim of the study was to compare colectomy rates for ulcerative colitis (UC) in SA to those of WE.* Methods*. Patients with UC were identified from a national administrative dataset (Hospital Episode Statistics, HES) between 1997 and 2012 according to ICD-10 diagnosis code K51 for UC. The colectomy rate for each ethnic group was calculated as the proportion of patients who underwent colectomy from the total UC cases for that group.* Results*. Of 212,430 UC cases, 73,318 (35.3%) were coded for ethnicity. There was no significant difference in the colectomy rate between SA and WE (6.93% versus 6.90%). Indians had a significantly higher colectomy rate than WE (9.8% versus 6.9%, *p* < 0.001). Indian patients were 21% more likely to require colectomy for UC compared with WE group (OR: 1.21, 95% CI: 1.04–1.42, and *p* = 0.001).* Conclusions*. Given the limitations in coding, the colectomy rate in this cohort was higher in Indians compared to WE. A prospectively recruited ethnic cohort study will decipher whether this reflects a more aggressive phenotype or is due to other confounding factors.

## 1. Introduction

Ulcerative colitis (UC) and Crohn's disease (CD) are chronic inflammatory bowel conditions, the cause of which is unknown. The prevailing hypothesis is of a dysregulated immune system in response to microbial and environmental triggers in genetically predisposed individuals [1].

The highest IBD incidence and prevalence rates are reported from North America and Europe [2]. In contrast, although the IBD incidence in developing nations was previously reported to be low, it seems to be increasing as these countries become more industrialised [3, 4]. Demographic changes occur due to increasing globalization and migration [5]. Previous studies showed that migrants develop the incidence of their adopted country [6–8]. Studying disease in migrant populations offers a unique opportunity to examine factors that impinge on new migrants resulting in disease presentation.

India, Pakistan, and Bangladesh rank highly within the commonest non-UK countries of birth. Two early studies examining migrants from these countries demonstrated a higher prevalence of UC in South Asians than Europeans [9, 10]. In the earlier study, during the 1980s, the standardised incidences of UC (10^5^ cases/year) in South Asians also differed within ethnic groups: 16.5, 10.8 and 6.2 in Sikhs, Hindus and Muslims respectively [9]. A later study reported on the incidence of UC in the Bangladeshi community in East London which was amongst the lowest in the world, with mean standardised incidence (10^5^ cases/year) of 1.8 compared with 10.8 in the South Asian group in Leicester over the same time period [11].

Moreover, the mean age at diagnosis was significantly lower in South Asian patients compared to Caucasians (26.5 years versus 33.5 years) [9]. Walker et al. studied the phenotype of UC in Northwest London; 63% of South Asian UC patients had extensive colitis compared with 42.5% of the White European cohort (*p* < 0.0001) [12]. Two Canadian paediatric studies in British Columbia have also demonstrated more extensive disease in the South Asian population; the most recent study by Carroll et al. demonstrated shorter symptom duration with a requirement for earlier escalation of therapy [13, 14].

These studies suggest that South Asians show an increased prevalence of UC with different UC incidence and prevalence rates between Indians and Bangladeshis. Pancolonic ulcerative colitis appears to be more common in South Asians compared with Northern Europeans but there are no studies describing disease severity across ethnic groups. Moreover, these studies were performed more than 10 years ago, in a local setting, and the majority were retrospective. It is not clear whether the higher prevalence of pancolonic disease translates into more aggressive disease. This may be captured as progression to colectomy which is indicated in cases refractory to medical treatment (acute severe colitis and chronic disease) or complicated disease presenting as dysplasia associated colitis.

The primary aim of the study was to examine colectomy rate in ethnic groups in the UC population. Our secondary aims were to examine age at colectomy, emergency versus elective indication for colectomy, and gender.

## 2. Materials and Methods

We performed a retrospective population based study using the Hospital Episode Statistics (HES) database. The HES database is an administrative dataset encompassing all hospital episodes in England. A hospital episode is defined by patient contact with the hospital by outpatient investigations and inpatient admissions. The dataset used was pseudoanonymised and contains all hospital episodes between 1 April 1997 and 31 March 2012. Diagnoses are coded using the World Health Organisation's (WHO) International Classification of Disease version 10 (ICD-10) and procedures are coded using the Office of Population Censuses and Surveys Classification of Surgical Operations and Procedures, 4th revision (OPCS-4).

### 2.1. Patient and Procedure Identification

Patients with a diagnosis of ulcerative colitis (UC) were identified using the appropriate ICD-10 diagnosis code for UC (K51). In instances where patients had episodes coding for Crohn's disease or unspecified colitis, the last admission code for inflammatory bowel disease (IBD) was regarded as reflective of the patient's true diagnosis.

The appropriate OPCS codes for subtotal and total colectomy and panproctocolectomy were used. Patient factors such as age, ethnicity, and type of admission (emergency or elective) were available. Dates of surgery and lengths of stay were calculated from given admission date, date of procedure, and discharge dates. Comorbidities were calculated using the Charlson Comorbidity Index, using their original weighting.

The main exclusion was patients without an ethnicity code. Patients who had a colectomy for cancer were removed from the analysis because we cannot differentiate between a colectomy for sporadic cancer and dysplasia. Therefore, only patients who had a colectomy for UC refractory to medical treatment were included.

The census from 2011 provided the total number of each ethnic group in England. The total number of each ethnic group was as follows: White 45,226,247 (85.3%), Indian 1,395,702 (2.6%), Pakistani 1,112,282 (2.09%), Black 1,846,416 (3.5%), Bangladeshi 436,514 (0.8%), and Chinese 379,503 (0.7%). We compared the census population with our coded cohort to examine whether the distribution of UC cases between ethnic groups reflected the background population.

### 2.2. Ethical Considerations

Ethical approval was sought and obtained from the National Research Ethics Service (NRES) committee (Project ID 134045, REC reference 13/LO/1235). Local research and development approval was obtained from London North West Hospitals Trust (RD reference 13.051).

### 2.3. Statistical Analyses

Descriptive statistics were used to describe ethnic groups and proportions. Nonparametric data were reported as medians with accompanying interquartile range (IQR).

Colectomy rates were calculated for each ethnic group as % of total number in group. Differences amongst patient groups were examined using chi-squared and Kruskal-Wallis test of a difference where appropriate. Binary logistic regression analyses were undertaken to examine factors associated with subtotal/total colectomy. Variables were initially tested independently to determine significant association with the outcome and those that reached significance of *p* ≤ 0.10 were considered significant and entered into a regression model. At multivariable analyses, variables that reached significance of *p* = 0.05 or less were deemed significant. All statistics were calculated using the Statistical Package for the Social Sciences (SPSS), IBM, version 20.0.

## 3. Results

### 3.1. Study Population

A total of 73,318 patients (35.3%) with UC admitted to hospital had an ethnicity code. Of these, 1,352 had CRC and were therefore removed. There were 35,087 (51.7%) females. The median age was 55 years (range: 2–104 years, IQR: 29 years). The commonest group was White Europeans followed by Indians, Pakistanis, Black, Chinese, and Bangladeshis (Table 1). UC patients of a South Asian background tended to be younger compared with the White European group: 10.9% in the White European group were under the age of 30 years compared with 18.5%, 28.9%, and 30.2% of the Indian, Pakistani, and Bangladeshi groups, respectively (*p* = 0.001). Across all groups, the majority of patients had no existing comorbidity (82.3%). The lowest rate of comorbidity was amongst the Pakistani population (3.0% had scored >1 on the Charlson comorbidity score). There were no statistically significant differences in terms of gender across the ethnic groups.

Comparing the census data to the UC population showed a higher proportion of White European UC cases (94.4% versus 85.3%, Table 2). Indians were represented almost equally (2.6% versus 2.7%). There were less than expected UC cases in the Pakistani and Bangladeshi ethnic groups.

### 3.2. Colectomy Population

The colectomy rate excluding cases that underwent colectomy for CRC was 6.9% (*n* = 5,044/73,318). The colectomy rate was the highest in the Indian group and was significantly higher than in White Europeans (9.8% versus 6.9%, *p* < 0.001, Table 3(a)). The colectomy rate in the Black population was almost identical to that in the Caucasian population (6.8% versus 6.9%, resp.). Although the colectomy rate was higher in the Chinese population (8.9%), this was not significant due to the overall small number of colectomies in the Chinese population [15].

### 3.3. Ethnicity Coding across Hospitals

As there was wide variation in ethnicity coding across centres with overall ethnicity coding of only 35.3% of UC hospital episodes, we performed a subgroup analysis of hospital trusts according to coding activity to determine whether the observed differences in colectomy rate were consistent in areas with higher coding. The number of cases was stratified into hospital trusts with greater than 40% and less than 40% ethnicity coding. We chose 40% as the minimum requirement for coding performance as a higher level would have made the numbers in the groups too small for meaningful analysis.

The cohort from hospitals with high coding practice showed that 1288/1910 (67.4%) patients of Indian origin who had a colectomy were in the greater than 40% subgroup (Table 3(b)). Within this group, the colectomy rate was in fact higher (10.9% versus 9.8%). There was also an increase in the White European group (7.1% versus 6.7%).

### 3.4. Total Colectomy Population

Of the 5044 patients undergoing colectomy, 4037 (73%) had elective and 1481 (27%) had emergency colectomy. The median colectomy age was 47 years (range: 5–91 years, IQR: 27 years). The median age at colectomy for the South Asian subgroups was significantly lower compared with White European patients (White European 48 years, Indian 39 years, Pakistani 35 years, and Bangladeshi 29 years, *p* < 0.001). There was no significant difference in age at colectomy when comparing White European, Black, and Chinese groups. Only 17.8% of White European patients undergoing colectomy were under 30 years of age compared with 26.6% of Indian patients and 30.8% of Pakistani patients (Table 4). In contrast, there was a significantly greater proportion of patients aged >60 years in the White European group (26.2%) compared with Indian (5.9%) and Pakistani (5.8%, *p* < 0.001) patients. There were no differences across the patient groups in terms of gender (*p* = 0.680), comorbidity score (*p* = 0.687), or whether patients had an elective or emergency colectomy (*p* = 0.498).

### 3.5. Risk Factors Associated with Colectomy

At univariable analysis to identify factors associated with colectomy, gender (*p* < 0.001), age (*p* = 0.013), comorbidity (*p* < 0.001), and ethnicity (*p* = 0.004) were significantly associated with colectomy (Table 4). When these factors were entered into a multivariable analysis, female gender was associated with reduced risk of colectomy (OR: 0.75, 95% CI: 0.71–0.79, and *p* < 0.001) (Table 5). Similarly, patients of advanced age (>60 years old) were 60% less likely to undergo a colectomy (OR: 0.40, 95% CI: 0.36–0.44, and *p* < 0.001) compared with younger patients (<30 years old). Indian patients were 21% more likely to require colectomy for UC compared with the White European group (OR: 1.21, 95% CI: 1.04–1.42, and *p* = 0.013) whereas Pakistani patients were 30% less likely to undergo colectomy (OR: 0.70, 95% CI: 0.53–0.93, and *p* = 0.014).

## 4. Discussion

We used this dataset to study colectomy rate in patients with different ethnic backgrounds attending hospitals in England with UC. Although the colectomy rate was almost identical between South Asians and White Europeans, the Indian subgroup were 21% more likely to require a colectomy for UC than White Europeans. This study raises the possibility of a relationship between ethnicity and colectomy rate for UC.

Early literature suggests that the extent of colitis dictates colectomy risk [15–17]. Later studies largely supported these findings except for studies on different ethnic groups in England. A higher incidence of pancolonic disease in a cohort of South Asians was associated with a lower colectomy rate when compared with Northern Europeans in one study (6.6 versus 10.6%) [12] and South Asians were significantly less likely to have a surgery than Europeans in a separate study [9]. These discordant observations maybe due to a number of reasons. The first reason is the relatively small number of colectomies compared to our national database (17 versus 211 colectomies in South Asians) in the study by Walker. Secondly, the study by Probert was carried out from 1972 to 1989 which predates the significant rise in second-generation South Asians diagnosed with pancolitis [7]. Thirdly, this study demonstrated differences in colectomy rate between the SA groups; the Pakistani ethnic group was significantly less likely to undergo colectomy than Indians. In Walker's study, there was no distinction within the SA population which may explain the overall lower colectomy rate. The HES database lacked data on the extent of colitis; therefore, we were not able to determine whether higher colectomy rate was associated with higher prevalence of pancolitis. However, the increased risk of colectomy associated with ethnicity is suggestive of a more aggressive disease phenotype.

Previous epidemiological studies looking at ethnicity and UC suggested genetic predisposition with change in environment results in a different phenotype in the migrant population [9]. Juyal et al. showed a possible genetic link in the first genomewide association study comparing UC susceptibility loci between North Indians and White Europeans [18]. It showed significant genetic heterogeneity between the two populations and three novel risk loci in the North Indian population were discovered. The study concluded that assessing genetic heterogeneity between the different populations in combination with varying environmental exposures might explain discordant findings across ethnic groups.

Cultural changes such as Westernisation of the diet may play an important role in the differences [19]. Investigators from Leicester showed that South Asian Hindu IBD patients have significantly altered their traditional diet [20]. Dietary changes adopted by South Asians may therefore act as a potential environmental stimulus in disease development.

Our study also noted that South Asian groups had a colectomy for UC at a younger age than White Europeans. Indian patients admitted with UC had a younger age demographic than White Europeans (54.4% versus 32.4% less than 45 years old). The lower median age at colectomy has implications. On the one hand, it may imply a more aggressive phenotype for all South Asian ethnic groups. Alternatively, it may reflect other confounding factors such as poor compliance with medical therapy and delay in diagnosis related to cultural matters particularly as South Asian patients were shown to have significantly higher concerns about 5-ASA treatments and higher nonadherence than non-Asian patients [21]. Nonadherence to 5-ASA medication in UC is an important predictor of disease relapse [22].

An early age of colectomy may also reflect a different phenotype related to different microbial composition. The composition of the gut microbiota differs according to geography [23]. De Fillipo et al. found that children from Burkina Faso had significantly higher faecal diversity than children in urban Florence [24]. Studies on gut microbiome diversity in Han Chinese both in Hong Kong and in Australia compared to Australian Caucasians showed different bacterial composition in healthy subjects between regions and between ethnicities within the same country [25]. Further studies on bacterial diversity and functional characteristics of the microbiome between South Asians and Caucasians may reveal a microbial profile that could explain the more aggressive disease phenotype.

## 5. Limitations

There are some limitations to our study. HES is an administrative dataset and is therefore inherently susceptible to a certain degree of coding error, although various audits suggest that coding is 90–93% accurate [26]. The incomplete recording of the ethnicity code presents a number of challenges in the interpretation of the findings. Only 35.3% of the overall hospital population had a code recorded for ethnicity but this still represents the largest study (73,318 cases) on the association between ethnicity and colectomy. Furthermore, our subgroup analysis demonstrated that, in hospitals with higher recorded ethnicity codes, the colectomy rate was higher in Indians suggesting that potentially the amalgamation of the results may be underestimating the colectomy rate for this subgroup rather than overestimating as might be construed by comparing the results to the previous study by Walker et al. As HES is a national dataset, the analysis conducted was retrospective and therefore prone to an element of information bias. Confounders such as medications, lifestyle behaviours such as smoking and diet, compliance with therapy, patient choice, and clinical decision-making are not available in the dataset and may have influenced the results.

The strengths of our study lie in the size of the dataset, which not only encompasses the largest cohort to date on colectomy rates in ethnic groups but also collates data over a long period of time (15 years) making it less likely that minor coding differences sway the results towards one patient group. We compared the distribution of the UC cases by ethnicity to census data. There was preferential coding of the White European population. The Indian population is well represented but there is underrepresentation of the Pakistani and Bangladeshi ethnic groups. Furthermore, our overall colectomy rate (6.9%) fits in with the variation reported by previous studies [13] although our study is cross-sectional in design rather than longitudinal. These comparisons suggest that even though we studied an ethnically diverse population, colectomy rates were reflective of other populations and thereby generalisable.

In conclusion, given the limitations in coding within this cohort, UC patients of Indian ethnic group may be significantly more likely to require colectomy at a younger age than White Europeans. Whilst this might reflect a more aggressive disease phenotype, prospective studies are needed to offer explanations on the role of genetics, diet, and diversity and function of the gut microbiota and to uncover fresh clues to the pathogenesis of IBD.

## Figures and Tables

**Table 1:** Patient demographics for patients with UC admitted to hospitals in England between 1997 and 2012.

		White European	South Asian			Others		*p*
			Indian	Pakistani	Bang	Black	Chinese	
		*n* (%)	*n* (%)	*n* (%)	*n* (%)	*n* (%)	*n* (%)	
Gender	Male	32844 (48.3)	980 (51.3)	413 (50.2)	67 (51.9)	468 (47.7)	106 (55.2)	0.250
	Female	35087 (51.7)	930 (48.7)	410 (49.8)	62 (48.1)	513 (52.3)	86 (44.2)	

Age	<30 yrs	7396 (10.9)	353 (18.5)	238 (28.9)	39 (30.2)	176 (18.0)	16 (8.3)	**<0.001**
	31–45 yrs	14901 (22.0)	695 (36.4)	312 (37.9)	38 (29.5)	335 (34.3)	37 (19.3)	
	46–60 yrs	17344 (25.6)	507 (26.5)	174 (21.1)	27 (20.9)	206 (21.1)	47 (24.5)	
	>60 yrs	28036 (41.4)	355 (18.6)	99 (12.0)	25 (19.4)	261 (26.7)	92 (47.9)	

Comorbidity (Charlson score)	No comorbidity	56484 (83.1)	1689 (88.4)	707 (85.9)	103 (79.8)	831 (84.7)	166 (86.5)	**0.010**
	1	7633 (11.2)	144 (7.5)	91 (11.1)	19 (14.7)	102 (10.4)	18 (9.4)	
	>1	3814 (5.6)	77 (4.0)	25 (3.0)	7 (5.4)	48 (4.9)	8 (4.2)	

Total		67931 (94.3)	1910 (2.7)	823 (1.1)	129 (0.18)	981 (1.4)	192 (0.27)	71,966

**Table 2:** Comparison of number of UC cases and census population by ethnicity.

	White European (%)	Indian (%)	Pakistani (%)	Bang (%)	Black (%)	Chinese (%)
Number of UC cases	67931 (94.3)	1910 (2.7)	823 (1.1)	129 (0.2)	981 (1.4)	192 (0.3)
Census population	45,226,247 (85.5)	1,395,702 (2.6)	1,112,282 (2.1)	436,514 (0.8)	1,846,416 (3.5)	379,503 (0.7)

**(a):** 

	White European	South Asian			Others		Total
		Indian	Pakistani	Bang	Black Afro-Caribbean	Chinese	
	*n* (%)	*n* (%)	*n* (%)	*n* (%)	*n* (%)	*n* (%)	
No colectomy	63,217 (93.1)	1,722 (90.2)	771 (93.7)	123 (95.3)	914 (93.2)	175 (91.1)	66922
Colectomy	4,714 (6.9)	188 (9.8)	52 (6.3)	6 (4.7)	67 (6.8)	17 (8.9)	5044^*∗*^
Total	67931	1910	823	129	981	192	71,966

^*∗*^ *p* ≤ 0.001, significant differences between colectomy rates across ethnic groups.

**(b):** 

	White European	South Asian			Others		Total
		Indian	Pakistani	Bang	Black Afro-Caribbean	Chinese	
	*n* (%)	*n* (%)	*n* (%)	*n* (%)	*n* (%)	*n* (%)	
No colectomy	38615 (92.9)	1094 (89.1)	518 (93.2)	44 (93.6)	455 (92.7)	34 (94.4)	40760
Colectomy	2944 (7.1)	134 (10.9)	38 (6.8)	3 (6.4)	36 (7.3)	2 (5.6)	3157
Total	41,559	1228	556	47	496	36	43,917

**Table 4:** Patient demographics for patients that underwent colectomy based on their ethnicity.

		White European	South Asian			Others		*p*
			Indian	Pakistani	Bang	Black	Chinese	
		*n* (%)	*n* (%)	*n* (%)	*n* (%)	*n* (%)	*n* (%)	
Gender	Male	2572 (54.6)	106 (56.4)	27 (51.9)	3 (50)	31 (46.3)	10 (58.5)	0.680
	Female	2142 (45.4)	82 (43.6)	25 (48.1)	3 (50)	36 (53.7)	7 (41.2)	

Age	<30 yrs	839 (17.8)	50 (26.6)	16 (30.8)	4 (66.7)	11 (16.4)	2 (11.8)	**0.007**
	31–45 yrs	1324 (28.1)	78 (41.5)	22 (42.3)	1 (16.7)	34 (50.7)	5 (29.4)	
	46–60 yrs	1313 (27.9)	49 (26.1)	11 (21.2)	1 (16.7)	13 (19.4)	3 (17.6)	
	>60 yrs	1234 (26.2)	11(5.9)	3 (5.8)	0	9 (13.4)	7 (41.2)	

Comorbidity (Charlson score)	No comorbidity	4274 (90.7)	176 (93.6)	46 (98.5)	6 (100)	61 (91.0)	16 (94.1)	0.687
	1	360 (7.6)	9 (4.8)	5 (9.6)	0	4 (4.0)	1 (5.9)	
	>1	80 (1.7)	3 (1.6)	1 (1.9)	0	2 (3.0)	0	

Admission type for colectomy	Elective colectomy	3381 (71.7)	147 (78.2)	41 (78.8)	4 (68.7)	46 (68.7)	14 (82.4)	0.498
	Emergency colectomy	1333 (28.3)	41 (21.8)	11 (21.2)	2 (33.3)	21 (31.3)	3 (17.6)	
	Total	4,714	188	52	6	67 (100)	17 (100)	

**Table 5:** Logistic regression model of predictors of colectomy for ulcerative colitis.

		Univariable	Multivariable		
			Odds ratio (OR)	95% confidence interval (CI)	*p*
Gender	Male	**<0.001**	1 (ref.)		
	Female		0.75	0.71–0.79	**<0.001**

Age	<30 yrs	**<0.001**	1 (ref.)		
	31–45 yrs		0.79	0.72–0.86	**<0.001**
	46–60 yrs		0.66	0.60–0.72	**<0.001**
	>60 yrs		0.40	0.36–0.44	**<0.001**

Comorbidity (Charlson score)	No comorbidity	**<0.001**	1 (ref.)		
	1		0.74	0.66–0.82	**<0.001**
	>1		0.36	0.29–0.45	**<0.001**

Ethnicity	Caucasian	**0.004**	1 (ref.)		
	Black Afro-Caribbean		0.87	0.68–1.12	0.28
	Indian		1.21	1.04–1.42	**0.013**
	Pakistani		0.70	0.53–0.93	**0.014**
	Bangladeshi		0.53	0.23–1.21	0.134
	Chinese		1.32	0.80–2.18	0.278
